# Prospective memory and executive functions in adults across the wider autistic spectrum

**DOI:** 10.1038/s41598-026-57092-2

**Published:** 2026-06-10

**Authors:** Daniela Nürnberg, Mareike Altgassen

**Affiliations:** Department of Psychology, Johannes Gutenberg University Mainz, Mainz, Germany

**Keywords:** Prospective memory, Executive functions, Wider autistic spectrum, Variability in cognitive profiles, Neuroscience, Psychology, Psychology

## Abstract

Prospective memory (PM) refers to the cognitive ability of remembering to execute an intended action in the future. Empirical evidence indicates reduced PM performance in autistic individuals. This study aimed to explore PM performance across the wider autistic spectrum, including individuals with diverse cognitive profiles, and to investigate the impact of executive functions on PM. Thirty autistic and 30 non-autistic adults (18–65 years), matched for age, gender and non-verbal abilities, took part in the study. Participants completed an event-based PM task, and three executive function tasks measuring planning, inhibition and generativity abilities. Overall, non-autistic adults performed better in the event-based PM task in comparison to autistic participants. Better executive functioning was associated with better event-based PM performance in both groups; the completion time of the planning task significantly predicted PM performance. Results confirm earlier findings of lower PM performance in autistic individuals and close links of executive functions and PM. Considering the significant impact of PM on day-to-day life, future studies should develop practical interventions to support PM (e.g., targeted training in planning abilities, the use of agendas, to-do lists or assistive technologies), especially for autistic individuals who are dependent on external aid.

## Introduction

Prospective memory (PM) is defined as the cognitive ability to recall and execute intended actions at a designated time in the future^1^. PM is required for a variety of everyday tasks, e.g., planning the next day’s grocery shopping, ensuring the laundry is removed from the washing machine following the cycle, or maintaining punctuality for scheduled appointments^2^. PM is comprised of multiple processes and phases^3^. Initially, the individual forms an intention for the future and stores it in episodic memory while being engaged in other ongoing tasks. For the execution of the previously made intention at the appropriate moment, concurrent ongoing activities must be inhibited, and the individual must switch to the prospective action and execute it as planned. Hence, PM tasks rely on several cognitive processes, such as attention, episodic memory and executive functions^4^. PM tasks are distinguished by the cue that indicates the appropriate moment to initiate the task. For event-based tasks, the intended action needs to be remembered and executed when a specific stimulus is presented (e.g., remember to purchase bread when seeing the bakery. For time-based tasks, the action has to be performed at a designated point in time or after a specific period of time has elapsed (e.g., remember to remove a cake from the oven after 60 min^5^). A fundamental aspect of PM tasks is that the execution of the intended action may not occur immediately after the formation of the intention, and that the PM task is embedded within an ongoing activity^1^. Hence, strong executive function and episodic memory abilities are critical for successful PM^4^. Individuals encountering difficulties with these cognitive functions are likely to experience challenges with PM. For instance, individuals with ADHD exhibit difficulties in executive functions^6^ and episodic memory^7^, and PM^8–10^. Similarly, individuals with schizophrenia have been shown to demonstrate reduced abilities in executive functions^11^, episodic memory^12^ and PM^13^. Comparably, lower performance in executive functions^6,14^ and episodic memory^15^ have been reported in autistic individuals, as well as difficulties with PM (for an overview see^2,4^). Autism is a neurodevelopmental disorder characterized by impaired social communication and interaction, coupled with restricted, repetitive interests, activities, or behaviors^16^. Autistic individuals vary in the degree to which they experience difficulties with cognitive, verbal, motor, social and adaptive skills. Some autistic individuals show an intellectual disability (ID) and limited language skills, whereas others do not^17^. Furthermore, some autistic individuals exhibit greater severity of autism core characteristics, less adaptive skills and often require extensive lifelong support compared to others^18^. While about one third of autistic individuals have an ID (IQ < 70^19,20^), and almost one quarter have borderline ID (IQ 70–85^21^), research in the field of autism has increasingly focused on autistic individuals without ID since about the 1980s^21–23^. The absence of research on autistic individuals with ID and their dependency on external assistance in daily living highlights the critical need for research in this domain.

Most laboratory studies show reduced time-based PM performance in autistic in comparison with non-autistic adults^2,4^, see Altgassen and colleagues^24^ and Williams and colleagues^25^ for a similar pattern in autistic children). For instance, Williams and colleagues^26^ tested 17 autistic adults and 17 chronological age (CA)- and mental age (MA)-matched control participants on a computerized PM task. Participants memorized words and made “yes” or “no” recognition judgments on whether they had seen the word before as the ongoing task. For the time-based task, participants were required to press a specific button at two-minute intervals. For the event-based task, they had to press a specific button whenever the word was a musical instrument. Autistic adults were outperformed by controls on the time-based PM task, while no significant group differences in performance were observed on the event-based PM task. Landsiedel and Williams^27^ examined 25 autistic and 23 non-autistic adults employing the same time-based computerized task as Williams et al.^26^. Consistently, their findings revealed that the autism group performed lower in time-based PM compared to non-autistic adults. Time-based PM tasks put greater demands on executive functions compared to event-based PM tasks, as in time-based PM, there is mostly no external cue to prompt retrieval of the intended action, and the participant must actively monitor and keep in mind the elapsing time^28^.

In addition to time-based PM, some studies found difficulties in event-based PM in autistic adults. For example, Altgassen et al.^29^ compared 25 autistic adults with 25 CA- and MA-matched controls in the “Dresden Breakfast Task”. Participants had to prepare breakfast with real objects following specific rules and time constraints. Controls outperformed autistic participants in event- and time-based PM tasks. Kretschmer and colleagues^30^ compared 27 autistic adults with 27 CA- and MA-matched controls in the “Virtual Week”, a computerized game that simulates tasks of daily living. Controls performed better than autistic adults on event- and time-based PM tasks. A subsequent Virtual Week investigation yielded a similar conclusion. Autistic adults performed lower than non-autistic adults in event- and time-based PM and significantly reduced in time-based compared to event-based PM^31^. A further study by Charlton and colleagues^32^ investigated self-reported PM abilities in autistic and non-autistic adults aged 40–83 years using the “Prospective and Retrospective Memory Questionnaire”. Autistic individuals reported significantly more PM difficulties than the control group, with a statistically significant decrease in reported difficulties with increasing age only in the autistic group.

However, not all studies observed difficulties in PM in autism. For instance, Groenman and colleagues^33^ found spared performance of autistic individuals in event- and time-based PM tasks, comparing 82 autistic and 111 non-autistic adults in the “Amsterdam Breakfast Task”, a computerized adaptation of the Dresden Breakfast Task, and lab-based naturalistic PM tasks. Similarly, Faustmann and Altgassen^34^ found no significant group effects when comparing autism and control participants in a computerized version of the Dresden Breakfast Task including time- and event-based PM tasks.

Taken together, most studies have found PM difficulties in autistic adults compared to age- and ability-matched non-autistic adults. However, to date all studies have examined autistic adults excluding those with an IQ < 70. Only one study by Sheppard and colleagues^35^ investigated individuals with diverse cognitive profiles and compared 14 autistic children with low and 14 autistic children with high support needs to 26 non-autistic MA-matched children. The results showed that autistic children with high, but not with low support needs, performed worse than non-autistic children in event-based PM tasks. Further research is required to explore whether autistic adults with high support needs encounter comparable challenges in PM to those experienced by autistic children with high support needs.

As executive functions play a key role in PM in the general population^3,36^ and in various clinical groups (e.g., autism: Brandimonte et al.^4,28^, ADHD^9^, schizophrenia^37^), the present study will investigate the relationship of executive functions and PM. Executive function is a broad term that comprises a range of cognitive abilities located in the prefrontal cortex. These abilities include for example problem-solving, planning, working memory, inhibition, cognitive flexibility, generativity and the initiation and monitoring of actions^14^. Research into executive function performance in autistic individuals with diverse cognitive profiles (including ID) has yielded inconsistent results, depending on which aspect of executive function was measured. For instance, there are reported difficulties regarding their planning abilities when compared to MA-matched controls^38–40^. However, no differences in inhibition have been identified^38,40,41^. The findings regarding working memory, mental flexibility and generativity are inconclusive depending on variations in the age range of the participants and the tasks employed in the testing process^38,40,41^. For instance, difficulties in generativity are reported in autistic children with ID^40^, but not in autistic adults with ID^38^.

A relationship between PM and executive functions has been consistently found in the general population, in both younger^36,42^ and older^43,44^ adults. No study has been conducted on autistic adults with diverse cognitive profiles regarding this relationship. Research findings on autistic individuals without ID predominantly indicate an association between executive functions and PM. For instance, reduced event-based PM performance was associated with more working memory difficulties in autistic children aged between 4 and 11 years^45^. Su and colleagues^46^ conducted a study in children with higher and lower autistic traits, ranging in age from 4 to 12 years. Their findings indicated that working memory was a significant predictor of time-based PM performance in the lower autistic traits group, while inhibition significantly predicted PM performance in the higher autistic traits group. Consistently, lower time-based PM performance was associated with lower working memory capacity in autistic adults with a mean age of approximately 31 years^26^. Similarly, Altgassen and colleagues^29^ reported that better time-based PM performance was associated with better switching and working memory performance in autistic children. Conversely, Williams and colleagues^25^ did not find any association between time-based PM and cognitive flexibility in autistic children (mean age approximately 10 years).

The objective of the present study was to examine PM performance across the wider autistic spectrum, including autistic individuals with diverse cognitive profiles, compared to CA- and non-verbal abilities matched controls. A better understanding of the relationship between executive functions and PM in autism could enable tailored support (e.g., training in planning, different types of reminders, calendars etc.), especially for autistic individuals who are dependent on external aid and have special needs. In line with previous research^29–31,35^, we expected autistic adults to show reduced PM performance as compared to non-autistic adults. A simplified PM task (i.e., the Token Task) was selected (used before in older adults^47,48^) to accommodate the expected variability in cognitive abilities in the target sample. Regarding executive functions, we expected autistic adults to show reduced planning performance^38,40^, but similar performance in inhibition^38,40,41^, and generativity^38^ as compared to non-autistic adults. Moreover, we hypothesized that PM and executive functions performance would be related in both groups. The better the performance in executive functions, the better the performance in PM^26,36,45,46^.

## Methods

### Participants

A total of 70 autistic and non-autistic adults were recruited. Ten participants (six autistic and four non-autistic participants) had to be excluded while testing due to severe cognitive or physical impairments which made task completion impossible. The final sample comprised 60 adults, including 30 with an autism diagnosis (24 males, 5 females, 1 diverse) as well as 30 non-autistic adults (25 males, 5 females). The mean age of the autism group was 36.0 and of the control group 36.8 (see Table 1). Autistic participants were recruited from residential groups and sheltered workshops for people with disabilities, through mailing lists and flyers, and control participants were additionally recruited through neighborhood apps. The following inclusion criteria were applied: participants were required to be aged between 18 and 65 years, to be native German speakers, and for those in the autism group, to have received a clinical diagnosis of autism. Exclusion criteria were severe psychiatric or neurological disorders such as schizophrenia, bipolar disorder, or neurological illnesses, the use of psychotropic substances (incl. psychostimulants) as well as being non-speaking. First, autistic participants were recruited. Subsequently, control participants were recruited with a focus on ensuring that the two groups were comparable in terms of age, gender, and education level. Finally, the control group was parallel to the autism group in terms of chronological age, gender, and non-verbal ability (as measured by the matrices reasoning subtest of the German edition of the Wechsler Adult Intelligence Scale-Fourth Edition; WAIS-IV^49^; see Table 1). Note, that consistent with the extent literature, autistic participants showed a greater variability in non-verbal and verbal ability scaled scores compared to non-autistic adults^50^, see Table 2, and Fig. 2 in the Appendix). Groups differed in terms of verbal abilities, with the control group exhibiting significantly higher verbal abilities in the vocabulary subtest of the WAIS-IV compared to the autistic group. Comorbid psychological disorders were assessed via external reports for participants with external guardianship and via self-reports for individuals without external guardianship. In the autism group, 36.6% indicated the presence of a comorbid psychological disorder, 36.7% did not indicate a psychological disorder, and 26.7% refused to respond. The most prevalent comorbid mental/neurodevelopmental disorders in the autism group were affective disorders (eight participants), followed by ADHD (five participants) and PTSD/anxiety disorders (four participants). In the control group, 20.0% reported a mental/neurodevelopmental disorder (four participants with affective disorders, one participant with ADHD and one participant with obsessive–compulsive disorder).

**Table 1:** Participants characteristics.

	Autism (*n* = 30) *M* (*SD*); Range	Controls (*n* = 30) *M* (*SD*); Range	*F* (*df*)	*η*^2^
Age	36.0 (12.2); 19–65	36.8 (12.4); 18–64	0.064 (1,58)	0.001
NVA scaled scores	7.1 (5.4); 1–19	7.1 (3.0); 1–12	0.001 (1,58)	0.000
VA scaled scores	5.6 (4.5); 1–13	7.7 (3.2); 1–12	4.301 (1,58)*	0.069
SCQ total score^1^	*n* = 23 25.4 (5.1); 17–35	/	/	/
SCQ stereotyped^1^ behavior	*n* = 28 6.3 (2.1); 2–9	/	/	/
DEX total score^1^	*n* = 28 34.2 (8.7); 19–58	/	/	/

NVA = Non-verbal ability; VA = Verbal ability; *p < 0.05; **p < 0.01; ***p < 0.001; ^1^There was some missing data for the questionnaires on autism severity and executive dysfunction.

**Table 2:** Distribution of non-verbal and verbal ability scaled scores.

	Autism (*n* = 30) NVA/VA	Controls (*n* = 30) NVA/VA
< = 4	13/17	9/6
5–9	8/4	14/12
> = 10	9/9	7/12

NVA = Non-verbal ability; VA = Verbal ability.

The study was conducted in accordance with the Declaration of Helsinki and was reviewed and approved by the ethics committee of the institute of psychology in Mainz. All participants, and in case of external guardianship, parents or caregivers provided written informed consent. Participants were compensated for taking part in the study (10 EUR per hour).

### Materials

#### Verbal abilities

The verbal abilities of the participants were measured using the vocabulary subtest of the German version of the WAIS-IV^49^. The vocabulary subtest is designed to assess individuals’ lexical knowledge and their ability to verbally express the meanings of words. The task becomes more challenging with each word presented. Raw scores are converted into scaled scores. Higher scores indicate better performance.

#### Non-verbal abilities

The matrices fluid reasoning subtest of the German version of the WAIS-IV^49^ was used to measure participants’ non-verbal abilities. Participants are requested to discover patterns within a given design. As the task progresses, the level of difficulty increases with each matrix. Raw scores are converted into scaled scores. Higher scores indicate better performance.

#### Autism severity

The German version of the Social Communication Questionnaire (SCQ^51^, FSK; Fragebogen zur sozialen Kommunikation, sozialen Interaktion und stereotypen Verhaltensweisen^52^) was used to assess autistic traits (lifetime and current behaviors). The SCQ comprises 40 items and represents an external assessment that is completed by parents or caregivers. The response procedure is dichotomous, employing a “yes” or “no” response. The first item is a screening question to determine whether the participant is speaking. For speaking participants, the initial 18 items evaluate the participant’s current behavior, and the final 21 items refer to their behavior at the age of 4–5 years. For non-speaking participants, several items are skipped and the questionnaire continues directly with item eight. The questions are classified into three categories: social communication (15 items), social interaction (14 items) and stereotypical behavior (nine items). One item belongs to no category. The maximum score is 39 points, with a standard cut-off score of 16 for a diagnosis of autism.

#### Executive dysfunction

The questionnaire of everyday and behavioral assessment of executive dysfunction (DEX) from the Behavioral Assessment of the Dysexecutive Syndrome (BADS^53^) was used. The DEX is a parent-rated questionnaire to measure executive functions in everyday life. The questionnaire comprises 20 items of three different categories, namely behavior, cognition and emotion. Everyday problems are rated using a 5-point Likert scale, ranging from “0 = never” to “4 = very often”, with intermediate options including “1 = rarely”, “2 = sometimes”, and “3 = often”. The total score is calculated by adding up the points, with a total sum score ranging from 0 to 80. The higher the score, the higher the executive dysfunction.

#### Prospective memory

PM performance was assessed with the “Token Task”^48^. Participants were shown a box and seven coins. They should always put a coin into the box whenever the experimenter started a new task instruction with the sentence, “Now, we’re going to do a task with pictures”. The procedure was first practiced, ensuring that participants understood the task. The sentence was said four times at fixed time points during the testing session. Participants received one point for correctly placing a coin into the box (maximum of four points) and were required to respond prior to the beginning of the subsequent task.

#### Executive function planning

**Fig. 1:**
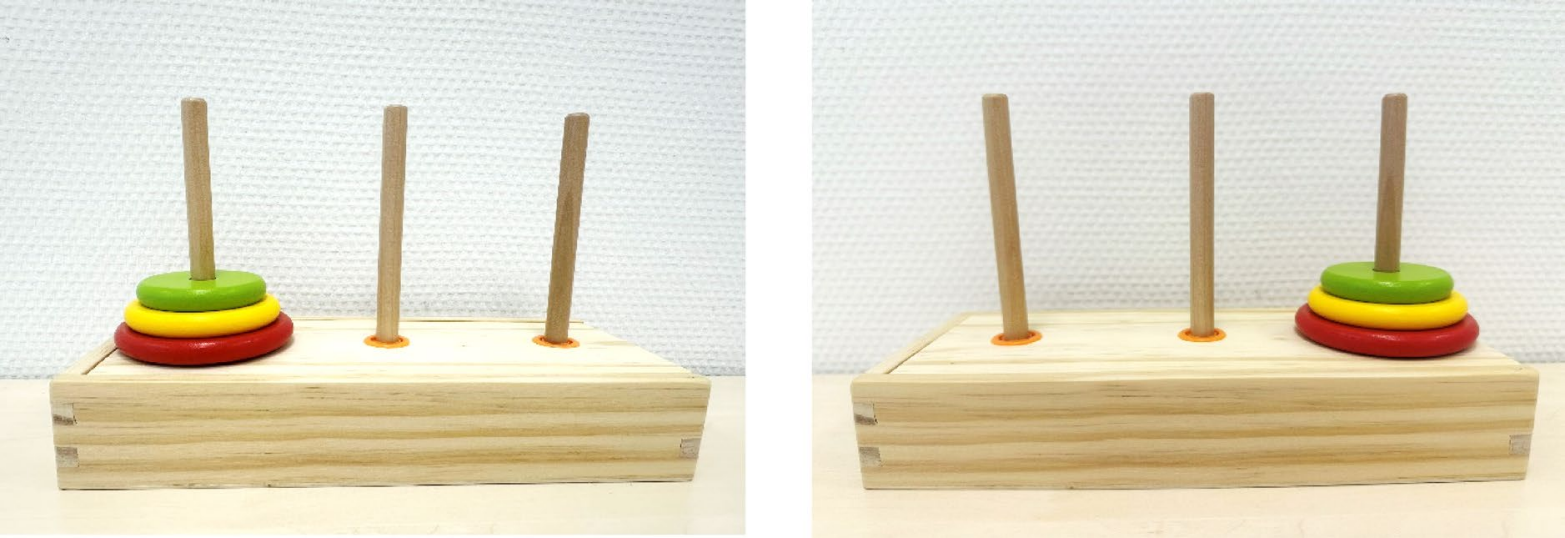
Tower of Hanoi. Start position on the left and the finish position on the right.

**Fig. 2:**
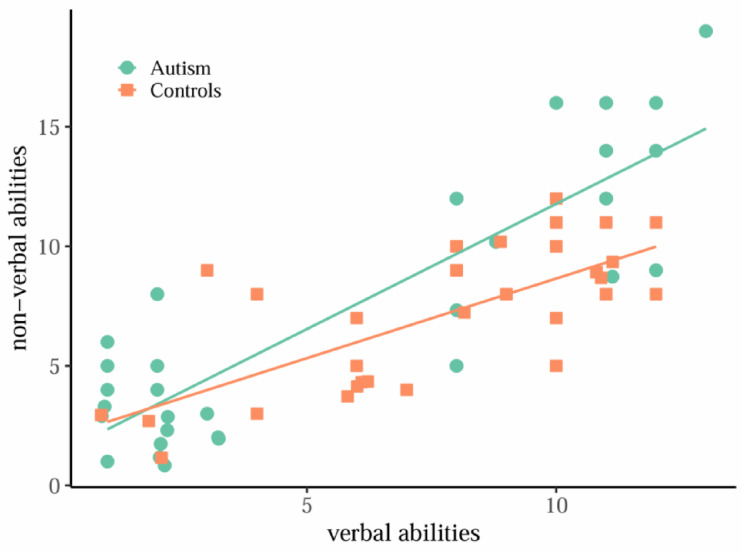
Scatter plot of verbal and non-verbal abilities for autistic and control participants. Note. Identical values are displayed slightly jittered.

Planning abilities were assessed with the “Tower of Hanoi”^54^. Participants were presented with a wooden model of three vertical bars and three discs of different sizes on the leftmost bar, with the largest disc at the bottom and the smallest disc at the top. The objective was to move the discs in the least possible number of moves to the rightmost bar (largest disc at the bottom, smallest disc at the top, see Fig. 1). Only one disc could be moved at a time, and a larger disc could never be placed on top of a smaller one. Participants initiated the experiment with a practice round involving two discs, subsequently proceeding to the main experiment involving three discs. In the case of successful completion, the task was repeated with four discs. Dependent measures included the number of moves and the time required to complete the task (in seconds). The fewer the number of moves and the faster the task was solved, the better the performance.

#### Executive function inhibition

Inhibition was assessed using a computerized version of the “Go-NoGo” test^55,56^. Participants were instructed to press the G-key in response to the presentation of a “heart” symbol and to refrain from responding to the presentation of a “circle” symbol. Items were presented for a duration of 2000 ms with an interstimulus interval of 250 ms. Twenty-five percent of all stimuli were NoGo trials. Participants were required to complete a practice block with feedback (2000 ms) consisting of 12 trials. Following this, two sets of 40 task trials each were conducted. In addition to the correct pressing of the G-key, the reaction speed of the participants was measured in seconds for Go trials. The more accurate the performance of the task, the better the overall performance. Dependent variables were the overall task performance measured as the range of correct Go and NoGo trials and the inhibition of the circle symbols (only correct NoGo trials).

#### Executive function generativity

Participants were instructed to state as many words as possible that start with the letter “S” within a time span of one minute (Phonemic Fluency). Subsequently, they were tasked with naming as many animals as possible within a one-minute timeframe (Categorial Fluency). The more terms are named, the better the performance.

### Procedure

Testing was conducted either at the laboratory of the local university or at the participant’s residence, contingent upon their preference. Compared to controls, autistic participants demonstrated a clear preference for home testing (17 autistic participants and four controls were tested at home, while 13 autistic participants and 26 controls were tested in the laboratory). Testing was carried out on a one-to-one basis, unless the participant requested the presence of a parent or caregiver. All tests were conducted by the same experimenter to increase comparability. A written timetable using simple language was placed on the table in front of the participant, showing a simplified outline of the entire testing session. Firstly, the consent form was signed by each participant, the experimenter and, in instances of external guardianship, also by the parents or caregivers. The participants or their caregivers answered questions about sociodemographic, comorbid disorders and medication use. This was followed by the explanation of the PM task. The subsequent step in the experiment involved the administration of the vocabulary subtest. This was followed by an assessment of the three different executive function tasks. Thereafter, the participants completed the matrices subtest. The SCQ and DEX were filled in by the parents/guardians during the testing session or online prior to the test session.

### Statistical methods

To compare performance in PM and executive functions between groups (autism, controls), univariate analyses of variance (ANOVA) were conducted. In the first ANOVA PM performance was used as a dependent measure. In the subsequent ANOVAs dependent measures encompassed the number of moves and the completion time of the Tower of Hanoi with three and four discs; the overall performance, the inhibition and the reaction time in the Go-NoGo test as well as the performance in phonemic and category fluency. As verbal abilities differed between groups, a separate analysis of covariance (ANCOVA) was conducted to control for verbal abilities where group differences were found (for a similar procedure, see^57^). Correlational analyses by Pearson were performed to identify possible associations between PM and executive functions. Furthermore, a multiple linear regression analysis was conducted for exploratory purposes, utilizing the findings (strongest correlations) from the correlation analysis.

## Results

### PM performance

A univariate ANOVA revealed a significant difference between the groups in PM performance, *F*(1,58) = 9.84, *p* = 0.003, *η*_p_^2^ = 0.145. Non-autistic adults performed significantly better in the Token Task, i.e., placing more coins in the box following the PM cue, compared to autistic participants (see Table 3). The significant group effect remained even after controlling for verbal abilities as a covariate, *F*(1,58) = 5.71, *p* = 0.020, *η*_p_^2^ = 0.091.

**Table 3:** Performance in PM and executive functions of both groups.

	Autism (*n* = 30) *M* (*SD*)	Controls (*n* = 30) *M* (*SD*)	*F(df)*	*η*^2^
PM (Token Task)	1.73 (1.93)	3.13 (1.50)	5.71 (1,58)*	0.091
ToH3 moves	11.25 (6.69)	10.92 (4.34)	< 1	
ToH3 time	79.58 (64.23)	57.96 (53.38)	1.67 (1,48)	0.034
ToH4 moves	29.67 (10.17)	25.86 (9.37)	1.35 (1,34)	0.038
ToH4 time	190.60 (119.11)	140.10 (101.24)	1.88 (1,34)	0.052
Go-NoGo^1^ ratio	0.88 (0.19)	0.92 (0.13)	< 1	
Go-NoGo inhibition ratio	0.92 (0.11)	0.88 (0.12)	1.96 (1,58)	0.033
Go-NoGo mean response time	0.61 (0.22)	0.49 (0.19)	5.74 (1,58)*	0.090
Phonemic fluency	10.23 (7.41)	13.20 (6.21)	2.82 (1,58)	0.046
Category fluency	16.37 (9.35)	20.57 (7.48)	3.69 (1,58)	0.060

ToH3 = Tower of Hanoi with three discs; ToH4 = Tower of Hanoi with four discs; ^1^overall performance; *p < 0.05; **p < 0.01; ***p < 0.001.

### Performance in executive functions

#### Planning

Regarding the Tower of Hanoi comprising three discs, analyses included 24 autistic and 26 control participants who successfully completed the task. There were no significant group differences regarding the number of moves to solve the tower, *F* < 1, as well as regarding the completion time in seconds, *F*(1,48) = 1.67, *p* = 0.200, *η*_p_^2^ = 0.034. With respect to the task involving four discs, the analyses included 15 autistic and 21 control participants. There were no significant group differences regarding the number of moves, *F*(1,34) = 1.35, *p* = 0.254, *η*_p_^2^ = 0.038, and the completion time of the task, *F*(1,34) = 1.88, *p* = 0.179, *η*_p_^2^ = 0.052 (see Table 3).

#### Inhibition

No significant group differences were found in the overall performance of Go and NoGo trials, *F* < 1 or in the inhibition performance (correct NoGo trials), *F*(1,58) = 1.96, *p* = 0.164, *η*_p_^2^ = 0.033. However, autistic participants reacted slower than non-autistic participants in response to Go-Stimuli, *F*(1,58) = 5.74, *p* = 0.02, *η*_p_^2^ = 0.090 (see Table 3). To address potential confounding effects of comorbid ADHD on inhibition, the analyses were additionally repeated, this time restricted to participants without ADHD. The ASD effect on response time remained significant in the ADHD-free subsample (F(1,52) = 7.79, *p* = 0.007, η_p_^2^ = 0.130), while accuracy showed the same non-significant pattern as in the full sample (F(1,52) = 2.33, *p* = 0.133, η_p_^2^ = 0.043). These results indicate that comorbid ADHD did not significantly influence inhibition performance in the present sample.

#### Generativity

No significant group differences were observed either in phonemic fluency, *F*(1,58) = 2.82, *p* = 0.098, *η*_p_^2^ = 0.046, or in category fluency,* F*(1,58) = 3.69, *p* = 0.060, *η*_p_^2^ = 0.060 (see Table 3).

### Correlation analyses

In the autistic group, a faster completion time in the Tower of Hanoi, better overall performance in the Go-NoGo test and better performance in phonemic and category fluency were associated with better PM performance. Furthermore, a higher externally reported score in the DEX was found to be associated with poorer performance in PM and category fluency. In the control group better performance in the Tower of Hanoi (moves and completion time), the Go-NoGo test as well as phonemic and category fluency were associated with better PM performance. Regarding cognitive abilities, better performance in verbal and non-verbal abilities were associated with better performance in PM and in all executive function tasks in both groups. In the autistic group, a higher total score in the SCQ was associated with poorer performance in PM and in verbal and non-verbal abilities (see Tables 4 and 5).

**Table 4:** Pearson correlations autistic participants.

	PM	ToH3 moves	ToH3 time	Go-NoGo	PF	CF	DEX Total	NVA	VA	SCQ Total
ToH3 moves	− 0.361									
ToH3 time	− 0.585**	0.693***								
Go-NoGo^1^	0.424*	− 0.724***	− 0.645***							
PF	0.673***	− 0.527**	− 0.680***	0.593***						
CF	0.577***	− 0.378	− 0.620**	0.542**	0.837***					
DEX Total	− 0.371*	− 0.002	0.093	− 0.228	− 0.365	− 0.458*				
NVA	0.790**	− 0.553**	− 0.716**	0.534**	0.754***	0.684***	− 0.215			
VA	0.872**	− 0.531**	− 0.670***	0.461*	0.777***	0.673***	− 0.392*	0.873***		
SCQ Total	− 0.435*	0.086	0.251	− 0.109	− 0.191	− 0.156	0.519**	− 0.412*	− 0.449*	
SCQ SB	− 0.171	0.290	0.201	− 0.306	− 0.180	− 0.323	0.428*	− 265	− 201	0.487*

ToH3 = Tower of Hanoi with three discs; ^1^overall performance; PF = phonemic fluency; CF = category fluency; NVA = non-verbal abilities; VA = verbal abilities; SCQ SB = SCQ stereotyped behavior; *p < 0.05; **p < 0.01, ***p < 0.001.

**Table 5:** Pearson correlations non-autistic participants.

	PM	ToH3 moves	ToH3 time	Go-NoGo	PF	CF	NVA
ToH3 moves	− 0.433*						
ToH3 time	− 0.543**	0.850***					
Go-NoGo^1^	0.652***	− 0.748***	− 0.893***				
PF	0.448*	− 0.526**	− 0.580**	0.647***			
CF	0.466**	− 0.542**	− 0.447*	0.557**	0.835***		
NVA	0.517**	− 0.541**	− 0.462*	0.471**	0.606***	0.617***	
VA	0.718***	− 0.602**	− 0.510**	628***	770***	0.773***	0.713***

ToH3 = Tower of Hanoi with three discs; ^1^overall performance; PF = phonemic fluency; CF = category fluency; NVA = non-verbal abilities; VA = verbal abilities; *p < 0.05; **p < 0.01, ***p < 0.001.

Regarding the multiple linear regression the overall performance in the Go-NoGo task, the completion time of the Tower of Hanoi with three discs, and the overall performance in fluency (phonemic and category fluency) were included as predictors. The dependent variable was the PM performance (Token Task). Only the completion time of the Tower of Hanoi with three discs was a significant predictor of PM performance (*p* = 0.007; see Table 6).

**Table 6:** Regression analysis, dependent variable performance in PM.

Predictors	*B*	*SE*	*β*	T	*p*
ToH3 time	− 0.015	0.005	− 0.524	− 2,823	0.007
Go-NoGo^1^	0.030	0.019	0.245	1.604	0.116
Overall fluency	− 1.601*	2.192	− 0.127	− 0.731	0.469

*N* = 50; *R*^*2*^ = 0.381; corrected *R*^*2*^ = 0.341; *F* (3,46) = 9.44, *p* =  < 0.001; ToH3 = Tower of Hanoi with three discs; ^1^overall performance.

## Discussion

In this study, we aimed to investigate PM performance across the wider autistic spectrum, including individuals with diverse cognitive profiles. PM and executive function performance were assessed in autistic and non-autistic adults.

### PM performance

As expected, a superior event-based PM performance in the Token Task was observed in non-autistic adults compared to autistic adults. These findings are consistent with the extent literature examining autistic adults without ID in event-based PM^29–31^, and with the only study that has investigated event-based PM in severely autistic children with minimal verbal ability aged between 5 and 13 years^35^. Individuals of both groups either responded within the specified timeframe (responding prior to the beginning of the subsequent task or did not respond at all. Furthermore, although all participants initially understood the task and were able to execute the Token Task correctly in the practice trial, at the end of the testing session some participants in both groups who were dependent on external aid were still able to recall that the coin had to be placed in the box, however, they could no longer recall the specific sentence upon which they should have done that. It is therefore possible that deficits in working memory or episodic memory contributed to their poor PM performance; this should be investigated by future studies. In general, the Token Task places relatively low demands on executive functions compared to other PM tasks that require interruption of ongoing activities or independent time monitoring^28,58^, and may therefore be suitable for individuals with ID if the working memory and episodic memory load imposed by the PM cue is reduced. Future studies could explore the potential benefits of simplifying the cue stimulus (e.g., by reducing the length of the sentence or using a single word) to reduce working memory and episodic memory demands. In addition, replacing the auditory PM cue (i.e., a specific sentence) with a simple visual cue may be beneficial, given the high prevalence of auditory processing difficulties in autism^59^ and the greater verbal difficulties observed in autistic individuals compared to non-autistic adults in the present study.

The present results suggest that autistic individuals may benefit from assistance in daily activities that require PM (e.g., planning, organizing or anticipating events or needs). Assistance may be provided in several ways, including the establishment of structure and routine, the utilization of reminders, timers or agendas, or specific training in organization or planning of daily routines^60–63^. A newer approach involves the integration of assistive technologies (e.g., intelligent assistants, virtual reality, planning applications) to enhance daily living and promote independence^64–66^.

### Executive function performance

As expected, autistic adults showed comparable generativity and inhibition performance to that of non-autistic adults^38,40,41^. In contrast to our hypothesis, our findings did not reveal any significant differences in planning abilities between autistic and non-autistic adults, although numerous studies examining autistic individuals with ID^38,40^ and without ID^67,68^ have documented reduced planning performance in comparison with non-autistic individuals. The absence of any group differences in the present study could be attributed to the difficulty level of the Tower of Hanoi task. For some participants, the task involving three discs was already too challenging, resulting in incomplete data. Consequently, the results of the tower with three, but especially with four discs, should be interpreted with caution. Moreover, the results showed a tendency for autistic participants to need more time to complete the tower tasks than non-autistic adults. This could be indicative of poorer planning skills; however, this effect did not reach statistical significance, which may be due to the low number of participants who had completed the task and the large variance of the data. Future studies should consider using the Tower of London task as a planning measure which has the option to select difficulty levels in smaller steps. The results highlight the importance of differentiating between executive functions, as autistic individuals can demonstrate full functionality in certain executive functions and difficulties in others. Importantly, the findings support the neurodiversity perspective, which emphasizes that autism should not be seen as a purely deficit-based condition^69^. Therefore, research should consider not only deficits, but also the strengths and diversity of cognitive profiles in autistic individuals.

### PM performance and executive functions

As expected, better executive function performance was associated with better PM performance in both groups. Hence, better planning, inhibition and generativity performances were associated with better PM performance in both groups. Furthermore, higher externally reported executive dysfunctions were linked to poorer PM performance in the autistic group. These findings are consistent with those of previous studies which demonstrated a relationship between executive functions and PM performance in autistic children^45,46^ and adults^26^ without ID as well as in non-autistic children^70–72^ and adults^73,74^. Longitudinal studies are needed to explore the causal relationships between executive function and PM performance in autistic individuals over time. The findings of the linear regression analysis indicated that across both groups the completion time of the Tower of Hanoi task significantly predicted PM performance. This suggests that effective planning skills are pivotal for optimal PM performance, which is in line with previous research in non-autistic adults demonstrating that better planning abilities can enhance PM performance^61,62,73^.

### Limitations and future research

Several limitations should be noted and addressed in future studies. First, the groups differed in their verbal abilities, which may have influenced the results. Accordingly, verbal ability was controlled for in analyses where group differences were observed, and the group effect remained significant. Future studies may consider using the Similarities subtest rather than the Vocabulary subtest, as it assesses more comprehensive verbal abilities^75^. Second, the inclusion of a working memory assessment could have clarified the potential relationship between Token Task performance and working memory and should be considered in future research. Third, to minimize testing time for this sensitive population, detailed autism diagnostic assessments and comprehensive cognitive assessment to measure ID were not conducted. Moreover, due to an expected lack of variability, the DEX and SCQ were not administered to non-autistic participants, although their inclusion could have provided additional insight. Future studies may therefore benefit from conducting more comprehensive assessments across multiple testing sessions.

Furthermore, the present sample included individuals with diverse cognitive profiles. To obtain a more accurate understanding of PM in autistic individuals with ID, future studies should restrict the sample to this population. To address this issue, barriers need to be removed that currently make the recruitment of autistic individuals with ID difficult. For instance, the increase of networking and support options could facilitate a more efficient outreach, and the provision of additional information to parents and guardians regarding the potential benefits of research would serve to enhance their interest in participating. Relatedly, there is a paucity of tasks specifically designed for autistic individuals with ID. In the present study, positive experiences were made with tasks that enabled adapting difficulty level to the individual being evaluated (e.g., vocabulary and matrices test), addressing the wide variation in autistic individual performance levels. The development of such tasks should be prioritized in future research, particularly in view of the increasing heterogeneity observed in the autistic population^23^. Furthermore, subsequent studies could investigate the impact of the stimuli employed (e.g., visual versus auditory) on performance outcomes.

## Conclusion

Taken together, a group of individuals across the wider autistic spectrum, including diverse cognitive profiles, exhibited difficulties in event-based PM when compared to CA- and non-verbal ability matched controls. No significant differences were observed between the groups in the measured executive functions (planning, inhibition, generativity). Superior performance in executive functions was associated with superior PM performance in both groups, and the completion time of the Tower of Hanoi task significantly predicted PM performance in both groups. The results of the study indicate that autistic individuals may benefit from external support in daily activities that require PM, such as the use of reminders, assistive technologies or targeted training in daily routines. Moreover, the training of planning skills could be beneficial for PM performance (for instance, the repeated articulation of intentions, the decomposition of complex tasks into sub-steps, or the utilization of aids such as calendars or to-do lists). Future research should focus on the practical and everyday implementation of support options in PM for autistic individuals, especially for those who are dependent on external aid, a part of the spectrum that is often overlooked.

## Data Availability

The dataset analyzed during the current study is not publicly available due to the sensitive nature of the research and because participants did not give written consent for their data to be shared publicly, but the dataset is available from the corresponding author on reasonable request.

## Appendix

See Fig. 2.
